# Predictors associated with an increase in daily steps among people with prediabetes or type 2 diabetes participating in a two-year pedometer intervention

**DOI:** 10.1186/s12889-024-18766-6

**Published:** 2024-05-11

**Authors:** Kristina Larsson, Jenny Rossen, Åsa Norman, Unn-Britt Johansson, Maria Hagströmer

**Affiliations:** 1 Department of Health Promotion Science, Sophiahemmet University, Box 5605, Stockholm, 114 86 Sweden; 2 Department of Clinical Neuroscience, Karolinska Institutet, Stockholm, Sweden; 3 Department of Clinical Science and Education, Karolinska Institutet, Södersjukhuset, Stockholm, Sweden; 4 Department of Neurobiology, Care Sciences and Society, Division of Physiotherapy, Karolinska Institutet, Stockholm, Sweden; 5 Academic Primary Care Center, Region Stockholm, Stockholm, Sweden

**Keywords:** Intervention, Prediabetes, Response, Steps, Type 2 diabetes

## Abstract

**Background:**

This study aimed to explore predictors associated with intermediate (six months) and post-intervention (24 months) increases in daily steps among people with prediabetes or type 2 diabetes participating in a two-year pedometer intervention.

**Methods:**

A secondary analysis was conducted based on data from people with prediabetes or type 2 diabetes from two intervention arms of the randomised controlled trial Sophia Step Study. Daily steps were measured with an ActiGraph GT1M accelerometer. Participants were divided into two groups based on their response to the intervention: Group 1) ≥ 500 increase in daily steps or Group 2) a decrease or < 500 increase in daily steps. Data from baseline and from six- and 24-month follow-ups were used for analysis. The response groups were used as outcomes in a multiple logistic regression together with baseline predictors including self-efficacy, social support, health-related variables, intervention group, demographics and steps at baseline. Predictors were included in the regression if they had a *p*-value < 0.2 from bivariate analyses.

**Results:**

In total, 83 participants were included. The mean ± SD age was 65.2 ± 6.8 years and 33% were female. At six months, a lower number of steps at baseline was a significant predictor for increasing ≥ 500 steps per day (OR = 0.82, 95% CI 0.69–0.98). At 24 months, women had 79% lower odds of increasing ≥ 500 steps per day (OR = 0.21, 95% CI 0.05–0.88), compared to men. For every year of increase in age, the odds of increasing ≥ 500 steps per day decreased by 13% (OR = 0.87, 95% CI 0.78–0.97). Also, for every step increase in baseline self-efficacy, measured with the Self-Efficacy for Exercise Scale, the odds of increasing ≥ 500 steps per day increased by 14% (OR = 1.14, 95% CI 1.02–1.27).

**Conclusions:**

In the Sophia Step Study pedometer intervention, participants with a lower number of steps at baseline, male gender, lower age or higher baseline self-efficacy were more likely to respond to the intervention with a step increase above 500 steps per day. More knowledge is needed about factors that influence response to pedometer interventions.

**Trial registration:**

ClinicalTrials.gov, NCT02374788.

## Background

The prevalence of prediabetes and type 2 diabetes is increasing globally causing major economic consequences and individual suffering. Preventing and treating the disease is an area of importance [1]. It is well known that regular physical activity is linked to control and prevention of the disease [2] and can improve glycaemic control and cardiovascular disease complications [3]. A non-linear dose-response association exists between moderate-to-vigorous-intensity physical activity and mortality in people with type 2 diabetes [4].

All adults should be physically active with at least moderate intensity for 150–300 min per week, perform strength training at least twice a week and limit sedentary time, according to the current recommendations [5]. However, most people with prediabetes or type 2 diabetes do not follow this recommendation [6–8]. One way to reach this patient population is through primary care as an arena [9], and through interventions using pedometers as a motivational tool to increase physical activity [10–15]. Pedometer-based interventions could have an effect on daily steps for people with prediabetes or type 2 diabetes on a group level [12, 15]. However, individual variations can occur, and it can be helpful for primary health care professionals to know which individuals are most prone to benefit from a pedometer-based intervention. It is known that individual factors, such as age, gender and health status, can influence physical activity [16], but more knowledge about other factors (e.g., behavioural or demographical) that can influence response to interventions is needed.

The Sophia Step Study was a two-year, three-armed, pedometer-based intervention for primary care aiming to support individuals with prediabetes or type 2 diabetes to increase their daily number of steps and physical activity [17]. The effect of the intervention has been evaluated previously. On a group level, a trend towards an effect on moderate-to-vigorous-intensity physical activity and decreased time in sedentary behaviour was observed [18]. To reach individualised interventions within primary health care, predictors associated with response to interventions on physical activity need to be evaluated. Therefore, the aim of this study was to explore predictors associated with intermediate (six months) and post-intervention (24 months) increases in steps among people with prediabetes or type 2 diabetes participating in a two-year pedometer intervention. Findings from the current study could contribute to individualise physical activity interventions in the primary health care for people with prediabetes or type 2 diabetes.

## Method

### Study design and population

This study is a secondary analysis of people with prediabetes or type 2 diabetes from two interventions arms of the randomised controlled trial Sophia Step Study [17], for which data were collected between 2013 and 2020. Diabetes specialist nurses recruited participants from two urban and one rural primary care centre in Sweden. Participants were randomised to one of the two intervention groups or to the control group via sealed envelopes. The control group was not included in this study. The inclusion criteria were 40–80 years of age, HbA1c > 39 mmol/mol or fasting glucose > 5.6 mmol/l, and fluency in the Swedish language. Exclusion criteria were patients with newly prescribed insulin (< 6 months), myocardial infarction in the past six months, suffering from repeated hypoglycaemia or severe hypoglycaemia in the past 12 months, diabetic foot ulcer or risk of ulcer (severe peripheral neuropathy), serum creatinine > 140 mmol/l, other disease prohibiting physical activity, classified as being very physically active assessed by the Stanford Brief Activity Survey [19] and those with no access to the internet. Prior to participation, all participants signed a written informed consent form. The study was approved by the Swedish Ethical Review Authority in Stockholm (Dnr.2012/1570‑31/3) and complied with the Declaration of Helsinki.

### Intervention

The two-year intervention was developed for primary health care to support individuals with prediabetes or type 2 diabetes to become regularly physically active. A multi-component intervention group (*n* = 64) was offered a pedometer to self-monitor their daily steps and register them using an online platform, as a motivational component (the pedometer data was not included in the current study). They were also offered both group and individual counselling. The counselling was most intense during the first year (ten group and eight individual sessions) compared to the second year (two group and two individual sessions). A single-component intervention group (*n* = 59) was offered a pedometer for self-monitoring and registration of daily steps. A third group was a control group (*n* = 65) receiving care as usual. The control group was not included in this study. Details of the intervention and the data collection have been published previously [17].

### Measurement of steps

The number of daily steps was measured with the ActiGraph GT1M accelerometer (ActiGraph, Pensacola, FL). Participants wore the accelerometer on the lower back [20] during waking hours for seven consecutive days at zero, six and 24 months. The participants also noted their daily wear time in a diary which was used to confirm wear time and number of valid days. Participants were included if they had ≥ 3 days out of 7 days, and ≥ 10 h per day of valid wear time.

Cardiovascular morbidity and mortality in inactive individuals can be significantly reduced with an increase of 500 steps per day [21]. Therefore, to capture response to the intervention, an increase of 500 steps per day was chosen. A dichotomous variable was created: ≥500 increase in steps or < 500 increase or decrease in steps. Participants were included if they had data on one or two of the follow-up measurement points (six and/or 24 months).

### Measurement of included baseline predictors

Demographics (age, gender and university education) and data on health conditions (prediabetes or type 2 diabetes, and comorbidities) were collected using a questionnaire (self-reported in paper or digital format as the participants preferred), and from patient medical records at baseline. Body mass index (BMI) was measured at an initial visit with the diabetes specialist nurse. At baseline, the participants responded to a questionnaire including the following measurements.

### HADS

Depression and anxiety were measured with the Hospital Anxiety and Depression Scale (HADS), which is a useful indicator of the possibility of depression and anxiety [22]. This was a 14-item questionnaire with two subscales for depression and anxiety, with seven items for each subscale. It was based on a 4-point Likert response scale ranging from 0 to 3. The total score ranged from 0 to 21 for the depression scale and from 0 to 21 for the anxiety scale. A cut-off value of ≥ 11 was used to identify the presence of possible anxiety disorder and/or a risk of depression [23]. HADS has been shown to perform well for assessing both presence of anxiety disorders and the symptom severity in both primary care patients and the general population [24].

### EQ-VAS

Health-related Quality of Life was measured using one item, the EQ-VAS, from the EQ-5D 3 L questionnaire [25]. Approval to use EQ-5D-3 L was received from the EuroQol Group. EQ-VAS measures overall health status on a vertical visual analogue scale ranging from 0 to 100, where 0 indicates the worst imaginable health and 100 the best imaginable health.

### Social support for exercise

Social support for exercise was measured using the Physical Activity Social Support questionnaire [26]. This was based on a 4-point Likert response scale (1 = strongly agree to 4 = strongly disagree) and addressed general support (one item), support from friends (two items) and support from family (two items). It was scored by dichotomising each question (1–2 recoded to 1 = support, 3–4 recoded to 0 = no support) and creating a sum score from 0 (low support) to 5 (high support).

### Self-efficacy for exercise

Self-efficacy for exercise was measured with the Self-Efficacy for Exercise Scale [27]. This was based on a 7-point Likert response scale (1 = not at all confident to 7 = very confident) and addressed confidence in continuing to exercise when feeling tired, being in a bad mood, not having time, being on vacation and in the event of bad weather. A sum score of all sub-items ranging from 5 (low self-efficacy) to 35 (high self-efficacy) was used as the outcome.

### Statistical analysis

Analyses were conducted in IBM SPSS version 27.0. First, bivariate analyses explored which baseline predictors to include in regression models. Chi-square was use for categorical variables and independent t-test for continuous variables. Predictors with a *p*-value < 0.2 were included in the regression models [28]. Second, multiple logistic regression models explored the odds and related 95% confidence intervals for associations of predictors at baseline with intermediate (six months) and post-intervention (24 months) changes in steps.

## Results

**Fig. 1:**
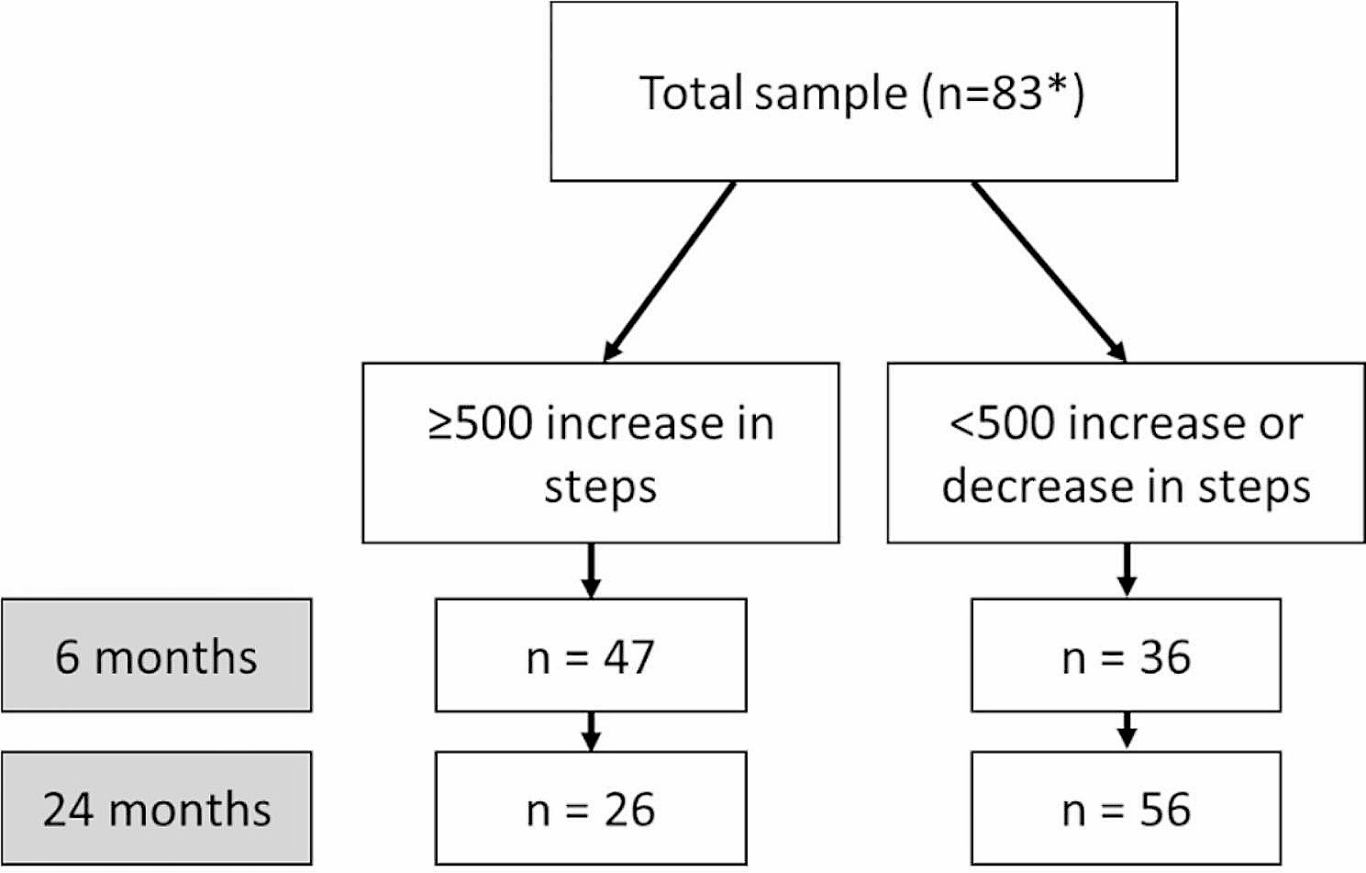
Flow chart of number of participants in each group at six and 24 months. *At 24 months the total sample were 82 participants

In total, 83 participants were divided into the step groups of ≥ 500 and < 500 steps, according to their response to the intervention. Figure 1 shows the number of participants in each response group. Table 1 describes the baseline characteristics of the sample. Overall, 16% of the participants had prediabetes: mean (± SD) age was 65.2 ± 6.8 years, 33% were female and 41% had a university education. Descriptive characteristics of the total sample at baseline showed that HADS identified few participants with a tendency for anxiety and/or depression, and HADS was therefore not included in the bivariate or regression analysis. Tables 2 and 3 show the results from the bivariate analyses at six and 24 months respectively. Table 4 shows results from the multiple logistic regressions at six and 24 months.

**Table 1:** Descriptive characteristics of the total sample at baseline

Variable	Total sample (*n* = 83)
Female	27 (32.5)
Age in years	65.2 ± 6.8
University education	34 (41)
Prediabetes	13 (15.7)
Comorbidity, number	2.1 ± 1.0
HADS, No problems	71 (94.7)
HADS, Tendency for anxiety and/or depression	4 (5.3)
BMI	29.6 ± 4.2
Self-efficacy	22.4 ± 6.8
Social support	3.4 ± 1.4
EQ-VAS	74.5 ± 16.3
Number of daily steps all participants	6568 ± 3073
Number of daily steps female	6794 ± 3403
Number of daily steps male	6433 ± 2880
Intervention randomisation group:	
Multi-component group	40 (48.2)
Single-component group	43 (51.8)

HADS = Hospital Anxiety and Depression Scale, BMI = Body Mass Index, EQ-VAS = Health-related Quality of Life. Continuous variables are presented as mean ± SD and categorical variables as n (%).

**Table 2:** Results from bivariate analysis at six months

Predictor		≥ 500 increase in steps	< 500 increase or decrease in steps	*p*-value between groups
Gender	Women, n	15	12	0.891
	Men, n	32	24	
Age in years	Mean (n)	65.6 (47)	64.8 (36)	0.575
University education	No, n	24	18	0.912
	Yes, n	19	15	
Diagnose	Prediabetes, n	5	8	0.150
	Diabetes, n	42	28	
Comorbidity physical	0–1, n	9	13	0.083
	≥ 2, n	38	23	
BMI	Mean (n)	29.9 (47)	29.1 (36)	0.360
EQ-VAS	Mean (n)	75.6 (42)	73.0 (33)	0.495
Self-efficacy	Mean (n)	23.1 (36)	21.7 (31)	0.411
Social support	Mean (n)	3.1 (33)	3.8 (42)	0.042
Steps at baseline	Mean (n)	5653 (47)	7763 (36)	0.002
Randomisation group	Multi-component group, n	17	23	0.877
	Single-component group, n	19	24	

BMI = Body Mass Index, EQ-VAS = Health-related Quality of Life.

**Table 3:** Results from bivariate analysis at 24 months

Predictor		≥ 500 increase in steps	< 500 increase or decrease in steps	*p*-value between groups
Gender	Women, n	7	24	0.166
	Men, n	19	32	
Age in years	Mean (n)	63.5 (26)	66.8 (56)	0.032
University education	No, n	11	30	0.335
	Yes, n	13	22	
Diagnose	Prediabetes, n	4	11	0.643
	Diabetes, n	22	45	
Comorbidity physical	0–1, n	7	12	0.583
	≥ 2, n	19	77	
BMI	Mean (n)	29.3 (26)	29.6 (56)	0.778
EQ-VAS	Mean (n)	78.9 (24)	72.4 (49)	0.107
Self-efficacy	Mean (n)	24.0 (22)	20.0 (42)	0.024
Social support	Mean (n)	3.4 (49)	3.3 (24)	0.795
Steps at baseline	Mean (n)	6982 (26)	6693 (56)	0.689
Randomisation group	Multi-component group, n	14	28	0.746
	Single-component group, n	12	28	

HAD = Hospital Anxiety and Depression Scale, BMI = Body Mass Index, EQ-VAS = Health-related Quality of Life.

**Table 4:** Odds of increasing ≥ 500 steps per day at six and 24 months, according to predictors at baseline

		OR (95% CI)
**6 months (** ***n*** ** = 75)**		
	Diagnose, diabetes	0.63 (0.14–3.13)
	Comorbidity, ≥ 2 diseases	0.40 (0.13–1.27)
	Steps at baseline	0.82 (0.69–0.98)
	Social support	1.33 (0.92–1.92)
**24 months (** ***n*** ** = 64)**		
	Gender, women	0.21 (0.05–0.88)
	Age	0.87 (0.78–0.97)
	EQ-VAS	1.01 (0.96–1.05)
	Self-efficacy	1.14 (1.02–1.27)

HAD = Hospital Anxiety and Depression Scale, EQ-VAS = Health-related Quality of Life.

Table 4 presents the odds of increasing ≥ 500 steps per day at six and 24 months, according to predictors at baseline. At six months, every 1000 increase in number of daily steps, the odds of increasing ≥ 500 steps per day decreased by 18%. At 24 months, women had 79% lower odds of increasing ≥ 500 steps per day, compared to men. For every year of increase in age, the odds of increasing ≥ 500 steps per day decreased by 13%. Also, for every step increase in self-efficacy, the odds of increasing ≥ 500 steps per day increased by 14%.

## Discussion

This is one of the first studies to explore predictors associated with intermediate and post-intervention increases in steps among people with prediabetes or type 2 diabetes participating in a physical activity intervention. The results showed that number of daily steps at baseline was a statistically significant predictor for increasing 500 steps per day or more at six months, and after two years the statistically significant predictors were male gender, lower age and higher self-efficacy.

At six months, responders to the intervention were more likely to have a lower number of steps at baseline. This is in line with a study showing that lower baseline physical activity level was a predictor for change in physical activity level over a six-month period of physical activity on prescription of treatment in people with metabolic risk factors [29]. Another study in a type 2 diabetes population found that larger increases in the number of daily steps at the beginning of the intervention period predicted an increase in physical activity at the eight-week follow-up [30]. However, these participants had a lower number of mean daily steps at baseline (4500 steps per day) in comparison to our participants, who were already active at the start of the intervention (mean 6500 steps per day).

The higher odds of men being a responder at two years might indicate that the support provided by the Sophia Step Study interventions may be better adapted to male participants. However, there might be other factors acting as barriers for women to increase their physical activity which we have not been able to capture. A meta-analysis concludes that for people with type 2 diabetes, common barriers for women to be physically active were lack of social support and motivation [31]. To capture these aspects, studies with a qualitative design might give further insights.

Responders to the intervention were younger, indicating that it might be more difficult to increase physical activity as people get older. Previous studies have found associations between increasing age and reduced physical activity levels [32–34]. Moreover, a review study by Choi et al. found that age was a negative predictor for participating in physical activity [35]. The results from the current study indicate that age-related factors seem to be associated with a response to the intervention. Older people could experience greater consequences of being physically inactive, such as sarcopenia, frailty and other chronic diseases, compared to younger people, making them a population on which there should be a particular focus in terms of finding supportive methods for increasing physical activity [36]. However, it is worth remembering that older people may have harder to adapt to technological methods, like mobile phone application. Also, as mentioned previously, the sample in the current study were already physically active at baseline, with mean daily steps of 6500. For our sample, it may be sufficient to maintain the current activity levels [37].

Participants with higher baseline self-efficacy for exercise were more likely to increase 500 steps or more over the two-year period. This is in line with a study investigating baseline predictive factors over a six-month period in people with metabolic risk factors who were prescribed physical activity, which found that higher baseline self-efficacy was correlated with change in physical activity levels [29]. Similar results were also found in a study with a population with chronic obstructive pulmonary disease, where higher self-efficacy at baseline predicted a positive response in a six-minute walk test [38]. Another study among middle-aged women also found that self-efficacy was an important predictor for physical activity [39]. As baseline self-efficacy appears to be an important predictor of physical activity, the importance of support to improve self-efficacy at the beginning of an intervention is emphasised. It has been shown that walking interventions with lengths ranging from eight weeks [40] up to 12 months [41] can improve self-efficacy levels. However, self-efficacy often acts as a mediator between the exposure (e.g., the intervention) and the outcome (e.g., physical activity) [42]. Studies in type 2 diabetes populations have shown that effects on physical activity after intervention with [43] or without pedometers [44] were mediated by self-efficacy. Since self-efficacy seems to be an important factor for being a responder to the Sophia Step Study, the results support conducting mediation analysis with self-efficacy in the future. Moreover, more research is needed to find supportive methods focusing on women, older participants and those with lower levels of self-efficacy, since they have lower odds of responding to this pedometer-based intervention.

It is known that health status can influence physical activity [16], however in the current study health conditions, like having prediabetes or type 2 diabetes or other comorbidities was not predictors associated with a response to the intervention.

The main strength of this study is the longitudinal design, which makes it possible to conduct an analysis evaluating predictors at baseline related to two-year measurements. Another strength is the use of the ActiGraph GT1M accelerometer, due to its validity for capturing steps at different walking speeds [45]. One limitation is the low number of participants, which may be a reason for not capturing other predictive factors that might be of importance for interpreting the data. Moreover, the results from this study can only be generalised to people similar to the current population in terms of high education level, high physical activity level and within the Swedish primary health care context. Another important aspect is the participants motivation to increase their physical activity, which might be higher compared to the general prediabetes or type 2 diabetes population, since they voluntary signed up to the study.

## Conclusion

In the Sophia Step Study pedometer intervention, participants with a lower number of steps at baseline, of male gender, of lower age or with higher baseline self-efficacy were more likely to respond to the intervention with a step increase above 500 steps per day. This study implies that it can be important to address level of self-efficacy for exercise early in an intervention, and to focus on improving self-efficacy at motivational sessions during the intervention period. This can be useful for health-care professionals when planning interventions considering the person-centred dialogue with people with prediabetes or type 2 diabetes.

## Data Availability

The datasets generated or analysed during the current study are not publicly available because data can be traced back to the study participants. According to Swedish and EU data legislation, access can only be granted upon a reasonable request. The request should be addressed to the PI and will be handled on a case‑by‑case basis. Any sharing of data will be regulated via a data transfer and use agreement with the recipient.

## Abbreviations

BMI: Body Mass Index
EQ-VAS: Health-related Quality of Life
HADS: Hospital Anxiety and Depression Scale
